# Describing the growth and nutritional status of sickle cell disease children and adolescents with reference to WHO growth standards in Cameroon

**DOI:** 10.1186/s40795-022-00650-4

**Published:** 2022-12-27

**Authors:** Eposse Ekoube Charlotte, Mbono Betoko Ritha Carole, Ida Penda Calixthe, Mony Elimbi Jeanne Georgette, Epee Patricia, Hassanatou Iyawa, Mandeng Ma Linwa Edgar, Budzi Michael Ngenge, Ntsoli Kofane Gaelle, Ekame Bulu Bianca Claudia, Sajida Misse Dicka, Koki Ndombo Paul, Kedy Mangamba Koum Daniele-Christiane

**Affiliations:** 1 Faculty of Medicine and Pharmaceutical Sciences, University of Douala, Douala, Cameroon; 2 Department of Pediatrics, Hopital Laquintinie Douala, Douala, Cameroon; 3 Faculty of Health Sciences, University of Buea, Buea, Cameroon; 4 Faculty of Medicine and Biomedical Sciences, University of Yaounde I, Yaounde, Cameroon

**Keywords:** Douala Laquintinie hospital, Stunting, Wasting, Underweight, Overweight, Sickle cell disease, Cameroon

## Abstract

**Background:**

Sickle cell disease (SCD) is a chronic disease with many complications among which is growth retardation. Here, we described the growth and nutritional status patterns of children with SCD and adolescents living in Douala, Cameroon.

**Methods:**

This cross-sectional study took place at the sickle cell treatment center of Douala Laquintinie Hospital from November 2015 to April 2016. The sociodemographic and anthropometric information of each SCD patient was determined, and then used for computing z-score indexes (weight for age, weight for height, body mass index for height, and height for age). The different indexes were used to determine the prevalence of malnutrition forms (stunting, wasting, underweight, and overweight/obesity) and compared to WHO standards by gender and age.

**Results:**

A total of 208 children and adolescents participated in the study. The mean age was 8 years (±5) and the median age was 7 years. Males accounted for 53.4% of cases, giving a sex ratio of 1:1.1. The proportions of wasting, stunting, underweight, and overweight/obesity in the overall population were 7.1% (*n* = 15), 9.1% (*n* = 19), 3.6% (*n* = 5) and 3.3% (*n* = 7) respectively. In children under 5, wasting, stunting, underweight, and overweight/obesity were noted in 1.4% (*n* = 1), 9.5% (*n* = 7), 1.4% (*n* = 1), and 5.4% (*n* = 4) respectively. In patients aged 5 years and above, a proportion of 10.5% (*n* = 14) was wasted, 9.0% (*n* = 12) were stunted, 5.9% (*n* = 4) were underweight and 2.2% (*n* = 7) were overweight/obese. The growth curve of children under five in our study was superimposable to the WHO standard growth curve. In children older than 5 years, the left shift for stunting was more pronounced for boys compared to girls.

**Conclusion:**

Nine percent of children and adolescents with SCD are stunted. The growth deficit appeared to be higher in patients aged 5 years and above, more particularly in boys than girls. Overweight/obesity was uncommon in our series. More robust research designs and statistical analyses are needed to confirm or refute these findings.

## Introduction

Sickle cell disease (SCD) was first described by JB Herrick in 1910 (Chicago, USA) by a black Jamaican student [1]. This disease is defined as a genetic disorder characterized by the presence in red blood cells of hemoglobin S **(**HbS)**,** an abnormal form of the protein used to transport oxygen [2]. Genes causing hemoglobinopathies are found in about 5% of the world’s population, and every year about 300,000 children are born with a major anomaly in hemoglobin - with more than two-thirds of them living in sub-Saharan Africa [3]. In Cameroon, it is estimated that nearly 18.2% and 2-3% of the population harbor SCD-related genes in their heterozygous and homozygous form, respectively [4].

SCD has multisystemic manifestations and is associated with significant morbidity and mortality [5]. Being a chronic disease, it affects the child’s growth drive and leads to growth retardation as reported previously in several settings such as Yemen, Tanzania, and The Democratic Republic of Congo [6–8]. Additionally, it was reported that socioeconomic status was the main factor influencing the growth and nutritional status of Nigerian patients with SCD [9]. Malnutrition, in theory, is a word that covers both under and over-nutrition. People are considered undernourished if the calories and protein they obtain from food are insufficient for their growth and maintenance, or overnourished if they ingest too many calories or are unable to utilize all of the nutrients in their diet owing to illness [10]. Stunting, wasting, being underweight, and micronutrient deficiencies are the four main ways in which undernutrition presents itself [11]. In Cameroon, few studies have been conducted on the growth and nutritional status of children with SCD [12, 13]. Ngo Um Sap et al. found a prevalence of underweight of 4% in children with SCD under 5 years of age [13].

Higher rates of metabolic expenditures in SCD people are thought to be a major driver of disease consequences owing to increased hematopoiesis, increased cardiac output, chronic inflammation, and other mechanisms [12]. Nutritional therapy has been recognized as an important feature of supportive management for SCD patients, particularly in resource-limited settings [12]. The paucity of studies addressing the growth and nutritional status patterns of children with SCD guided the realization of this study, especially in the Littoral region of Cameroon.

## Materials and methods

### Study design and study population

This cross-sectional study was conducted from November, 2015 to April 2016 at the sickle cell center of Douala Laquintinie hospital (DLH), Littoral region, Cameroon where approximately 600 patients are being followed yearly. It is the sole specialized center in the country where only sickle patients are been managed by a team of experts. All children and adolescents with laboratory-confirmed sickle cell disease aged 0 to 19 years who consulted or were admitted to the sickle cell treatment center at DLH were included in the study. Children and adolescents whose parents refused to participate in the study were excluded from the study. Assuming an estimated undernutrition prevalence in SCD of 4% in 2019 in Cameroon [9], the minimum sample size of 60 children was needed using Cochran’s formula [13]. To reduce selection and information biases, patients were enrolled consecutively, voluntarily, anonymously, and without remuneration. The refusal percentage was projected to be 20% of the sample. This made the final minimum sample size estimate to be 72 children.

### Procedures

From the registers of the service, we identified all the children with sickle cell disease followed at the sickle cell disease center of the DLH, and called the parents to explain the objectives of our study. We invited these parents and their children to an interview at the center and a pre-tested questionnaire was administered to those who agreed and signed their informed consent. Adolescents were also required to sign an assent form before enrolment. We then performed a physical examination and collected anthropometric parameters (weight and height) for all children and plotted these parameters on WHO gender-specific growth reference charts [14, 15]. Finally, we used the ANTHRO software to compare the growth of this population to normal growth according to WHO standards. The variables studied included socio-demographic characteristics (age, gender, educational level of children, level of education of parents, age at diagnosis of sickle cell, number of sicklers in the family) and anthropometric indices (weight-for-height index, height-for-age index, weight-for-age index, body mass index for age index).

### Anthro software

The standard Anthro software analyses four anthropometric indicators: length/height-for-age, weight-for-age, weight-for-length, weight-for-height and body mass index-for-age and is limited to children below 5 years. It has three components: the anthropometric calculator, the individual evaluation, and the nutritional survey. The first two modules are extremely relevant for clinical application since they deal with calculating z-scores (or percentiles) for the evaluation of each child’s growth. The WHO AnthroPlus software, which is used to track the development of school-age children and adolescents globally in accordance with the WHO Reference 2007, is available for children aged 5 to 19 years. AnthroPlus includes three relevant indicators (weight-for-age, height-for-age and BMI-for-age) to demonstrate consistency with the WHO Child Growth Standards for 0–5 years [16].

### Anthropometric measurements taken

#### Weight measurement

To weigh the children, we used an “OMRON” brand scale with the following characteristics: solid and resistant, electronic (digital readout), capacity of 150 kg, and precision of 0.1 kg (100 g). For a child under 2 years old or who was not able to stand, we performed the weighing by first weighing the mother alone and then weighing the mother carrying her undressed baby.

#### Height measurement

Depending on the child’s age and ability to stand, we measured their height when lying down (lying height) or when standing (standing height), using a horizontal or vertical measuring board that was handcrafted by a carpenter and placed on a stable surface (table or wall). For children under 2 years old, we measured their height while lying down. For children under 2 years old who refused to lie down to have their lying height measured, we measured their standing height and added 0.7 cm to convert this to lying height. For children 2 years or older who could not stand up, we measured lying down and subtracted 0.7 cm to convert their lying height to standing height.

### Anthropometric indexes [17]

- Height-for-age: it reflects the growth achieved in size lying or standing at the child’s age during a given visit. This indicator is used to identify growth retardation (stunting) which is defined as a z-score is < − 2. Severe stunting is considered when this z-score is < − 3
- Weight-for-age: It reflects body weight in relation to the age of the child at a specific date. This indicator is used to determine if a child is underweight or severely underweight. When the z-score is < − 2 we speak of underweight or low weight. Severe underweight is considered when this z-score is < − 3. Only patients less than 10 years of age were included in displaying this anthropometric index.
- Weight-for-height: it is an indicator of growth that relates weight and height in a lying or standing position, and indicates wasting (z score < − 2). It is also a useful indicator for detecting overweight (z score > 2) and obesity (z score > 3). BMI-for-age and weight-for-height lying/standing tend to give very similar results.
- BMI-for-age: To calculate a child’s BMI we use the following formula:
  $$BMI=\frac{\textrm{Weight}\ \left(\textrm{kg}\right)}{\textrm{Height}\ \left(\textrm{m}\right)\ \textrm{x}\ \textrm{Height}\ \left(\textrm{m}\right)}$$

The Body Mass Index (BMI) for Age is a figure derived from a child’s weight and height and assessed using an age- and sex-specific growth chart and serves as a trustworthy gauge of body fatness. This index was used for patients above 5 years to indicate wasting. Overweight was defined as Z-score between + 2 and + 3. Obesity was defined as a z-score > + 3. When using BMI indices, obesity was considered for any patient with a BMI above or equal to 30 kg/m2.

### Statistical analysis

The distribution curves were plotted using WHO Anthro software and AnthroPlus version 1.0.4 (for the sample population aged 5-19 years). This software gives WHO 2007 references for school-age children aged 5 to 19 years (z-scores) with confidence ranges and standard errors around the prevalence estimates. It has functions to compute prevalence estimates (and confidence intervals), z-scores, and summary statistics for z-scores (and confidence intervals). The Z scores are calculated using statistical software [18]. Results are given for age-appropriate height, weight, and body mass index indicators [19]. The primary outcome of choice was stunting and expected outcome was 10.5% as reported by Lukusa et al. [18] The following simple formula was used for calculating the adequate sample size for prevalence determination [20].

$$n=\frac{Z^2p\left(1-p\right)}{d^2}$$

Where n is the sample size, Z is the statistic corresponding to level of confidence(1.96 for 95% confidence interval), P is expected prevalence (10.5), and d is precision (0.05), the minimum sample size was 144 patients. For the evaluation of risk factors, a cohort design would have been more useful, as one would estimate from the same study [18], a percentage in non-exposed group of 9.8%, percentage in exposed group = 10.5%, with level of confidence at 95%, expected ratio between exposed and non-exposed = 1, difference in prevalence = 0.7%, and expected power = 80%, the minimum sample size for the exposed group would have 29,080 and 29,079 patients according to Kelsey and Fleiss respectively [21]. However, for the purpose, the scope and the potential weak power of the current study, *p*-values were not measured. Continuous variables were presented as mean ± standard deviation (SD) and categorical variables as frequency and percentage.

### Ethical considerations

This study was conducted following ethics directives related to research on humans in Cameroon. The study received ethical clearance from the Institutional Committee of Ethics for Research for Human Health of the University of Douala (N° CEI-UDO/301/12/2015/T) and, administrative agreement (N°5317/AR/MINSANTE/DHL/CM) was obtained from the Director of Laquintinie Hospital. Before enrollment and the administration of the questionnaire, parents and adolescents were informed of the purpose and process of the investigation and a signed informed consent was obtained from the children’s parents/guardians. Furthermore, adolescents (patients 10 years and above) without prejudice also provided their assent for the study. Respect for the confidentiality of the data collected throughout the study was ensured.

## Results

### Sociodemographic characteristics

In total we identified 224 patients, we excluded 16 (refused to participate in the study). Therefore 208 children and adolescents with SCD were included in the study. Sociodemographic characteristics are depicted in Table 1. Most patients were recruited in a steady state (*n* = 167, 80.3%) and only a few were recruited while being hospitalized (*n* = 41,19.7%).

**Table 1:** Sociodemographic characteristics of our population

Characteristics	Frequency	Percentage
**Gender**		
Male	111	53.4
Female	97	46.6
**Age group**		
0-5	74	35.6
5-10	65	31.3
10-19	69	33.2
**Educational level of children**		
None	27	13
Nursery	46	22.1
Primary	86	41.3
Secondary	47	22.6
Tertiary	2	1
**Number of sicklers in the family**		
1	176	84.6
2	22	10.6
3	5	2.4
> 3	5	2.4

The mean age was 8 years (±5). The age group of children under 5 years represented more than a third of the participants (*n* = 74, 35.6%). Boys accounted for 53.4% (*n* = 111) of patients giving a sex ratio of 1/1.1. The main educational level of the population was a primary school (*n* = 86, 41.3%) and only two adolescents (1%) were attending university. Concerning the educational level of parents, 87.7% of mothers and 89.5% of fathers attended at least secondary school. Families with at least two SCD patients represented 15.4% (of our study population when the majority of families (84.6%) had only 1 sickler.

### Anthropometric indices

In the overall population, the mean values of weight, height, and BMI was 26 (± 13.5) kg, 121 (± 26) cm, and 16.4 (± 2.7) kg/m^2^, respectively. The overall proportion of wasting, stunting, underweight and overweight/obesity were 7.1% (*n* = 15), 9.1% (*n* = 19), 3.6% (*n* = 9), and 3.3% (*n* = 7), respectively. When considering children under 5, wasting, stunting, underweight, and overweight/obesity were noted in 1.4% (*n* = 1), 9.5% (*n* = 7), 1.4% (*n* = 1), and 5.4% (*n* = 4) respectively as shown in Table 2. In children aged 5 years and above, a proportion of 10.5% (*n* = 14) was wasted, 9.0% (*n* = 12) were stunted, 5.9% (*n* = 4) were underweight and 2.2% (*n* = 7) were overweight/obese (Table 2). No patient had a weight for age < − 3 Z-score no matter the age. Most patients (93.5%, *n* = 130) had normal weight for their age (Z scores between − 1 < and < + 1). Patients with height for age < − 3 Z score accounted for 4.8% (*n* = 10) of the sample. Patients with weight for height > 3 Z score (indicating obesity) were found in only one patient (0.5%) and severe wasting (<− 3 Z score) in 4 patients (1.9%) (Table 2).

**Table 2:** Anthropometric index according to age groups

	Anthropometric index								
	Weight for Age (W/A)			Height for Age (H/A)			Weight for Height (W/H) and BMI for Age		
	Age groups in years (percentage %)								
	0-5	5-10	Total	0-5	5-19	Total	0-5	5-19	Total
**Z-score**									
< −3	0	0	0	6 (8.1)	4 (3.0)	10 (4.8)	0	4 (3.0)	4 (1.9)
< −2	1 (1.4)	4 (6.1)	5 (3.6)	1 (1.4)	8 (6.0)	9 (4.3)	1 (1.4)	10 (7.5)	11 (5.2)
< −1	15 (20.3)	13 (20.0)	28 (20.1)	11 (14.9)	44 (32.8)	55 (26.4)	5 (6.8)	24 (17.9)	29 (13.9)
0	47 (63.5)	40 (61.5)	86 (61.8)	48 (64.9)	65 (48.5)	113 (54.3)	47 (63.5)	84 (62.7)	131 (63.0)
> 1	7 (9.5)	9 (13.8)	16 (11.5)	5 (6.8)	12 (9.0)	17 (8.1)	14 (18.9)	9 (6.7)	23 (11.0)
> 2	4(5.4)	0	4 (2.8)	3 (4.1)	0	3 (1.4)	4 (5.4)	2 (1.5)	6 (2.8)
> 3	0	0	0	0	1(0.7)	1 (0.5)	0	1 (0.7)	1 (0.5)
**Total**	**74**	**134**	**208**	**74**	**134**	**208**	**74**	**134**	**208**

Km;k’;l

**Fig. 1:**
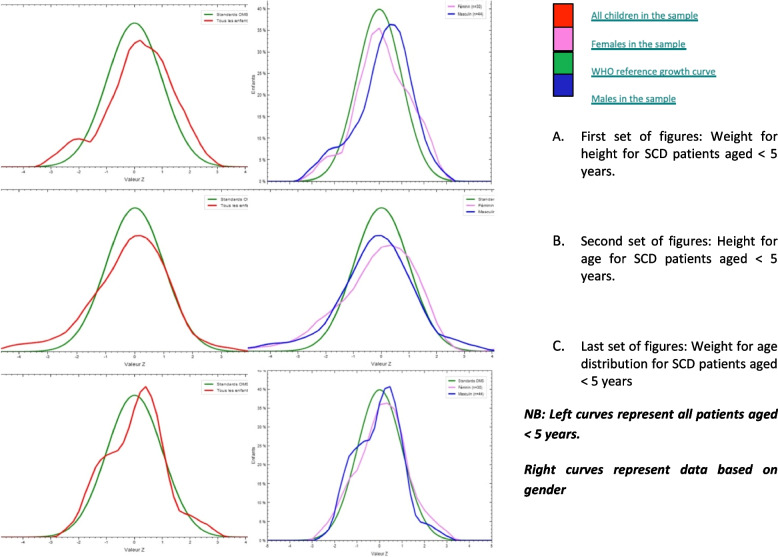
WHO growth curves for patients < 5 years compared to the SCD population by gender

When comparing the growth curves of underfives with WHO reference curves, we noted a shift in the curves weight-for-height index of both sexes towards the right limit. The weight-for-age and height-for-age index curves of both sexes were slightly shifted towards the left limit compared to the WHO reference curve as shown in Fig. 1.

**Fig. 2:**
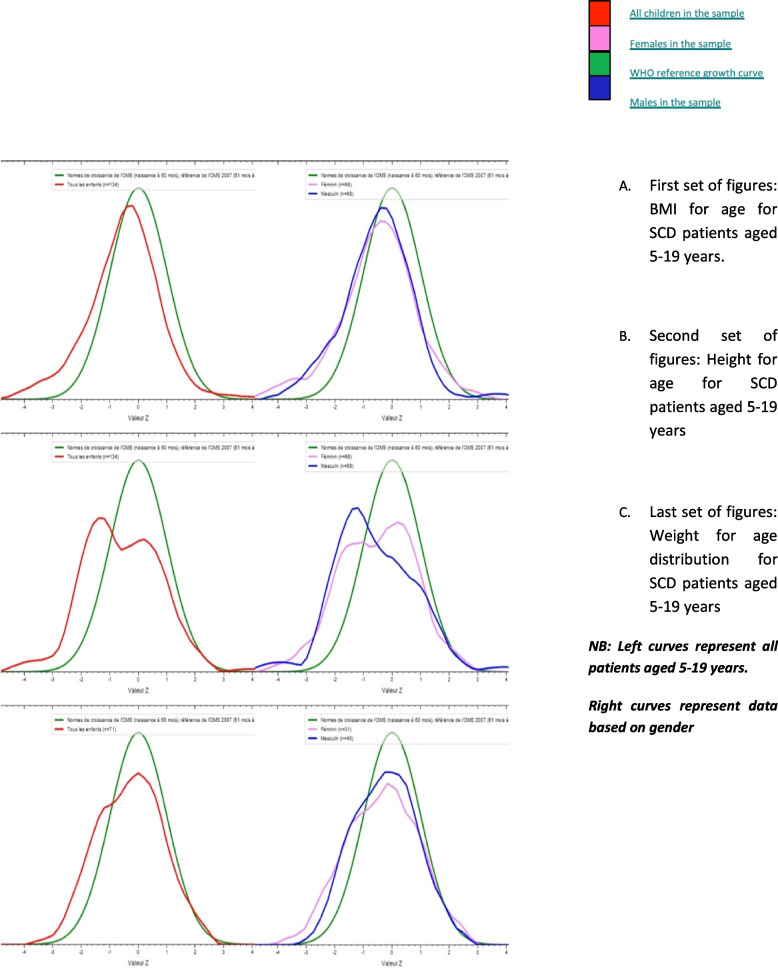
WHO growth curves for patients aged 5-19 years compared to the SCD population by gender

For children aged 5-19 years, we noted the shift of the two curves weight-for-age index of our population towards the left limit compared to the WHO reference curve and the median of the two curves was also lower than that of the WHO standard. We had a clear shift of the 2 curves height-for-age index of our population towards the left limit but more accentuated in the male sex than in the female sex compared to the WHO reference curve. We note that the 2 curves BMI for age had a clear shift towards the lower limit (left shift) compared to the WHO reference curve. The median for both boys and girls was lower than the WHO standard as shown in Fig. 2.

## Discussion

The objectives of our study were to describe the pattern of height and weight growth of children and adolescents with SCD followed or hospitalized at the SCD center at Douala Laquintinie hospital and compare it with the WHO growth standard. The WHO standards showed that the prevalence of global malnutrition was 12.8% [22]. Conversely, the National Center for Health Statistics reference showed a prevalence of global malnutrition at 23.1% [22]. The prevalence of undernutrition amongst children in a rural community in Cameroon has been reported to be 58.1% (23.6% were wasted, 26.5% were underweight and 49.9% were stunted) [23]. Comparing our results with other chronic illnesses like HIV denotes a rather better growth pattern in patients with SCD; Penda et al. found that the overall prevalence of malnutrition among HIV-infected children was 68.7%; 63.6% were stunted, 37.8% were underweight and 18.4% were wasted [24]. In urban settings, studies are sparse in Cameroon.

In our overall sample, a total of 9.1% were stunted, 7.1% were wasted, 3.6% were underweight and 3.3% were overweight/obese. A value of stunting closer to that in our sample was reported by Lukusa et al. [25] in Congo Kinshasa, Esezobor [26], and Ukoha et al. [27] in Nigeria who found respectively 10.5, 10.9% and, 11.6% of their sample. Another high prevalence (25.8%) of stunting was obtained from children in Ghana [28] and the USA (22%) [29]. But many authors found a much higher prevalence of wasting than us, ranging from 22 to 50% [25–27, 29]. This could be partly explained by the fact that the estimated prevalence of thinness by WHO among children and adolescents is lower in Cameroon compared with those of Congo and Nigeria (5.5% vs 9.9 and 9.7%) [30]. However, SCD and growth deficit have usually been captured in studies, with magnitude varying with geographical region. That can be explained by several mechanisms including chronicity of the disease leading to higher resting energy expenditure [31], repeated acute complications and hospitalizations [32], endocrine dysfunction [33], chronic anemia [34], and micronutrient deficiency [35]. Genetic, environmental factors, socio-economic conditions, and nutrition may also be part of the explanation.

In underfives, our prevalence of stunting was closer to the 4% reported by Ngo Um in Cameroon 3 years ago but much lower than the 19.5% proportion obtained by Shongo in the DRC in 2013 [8] and from a Nigerian study which reported 55.4% of cases as being stunted [26]. The difference with the Nigerian study is because it was a national survey while we did only a hospital-based study. We can presume that children coming to the hospital are followed up and have the minimum counseling to prevent sickle cell complications and growth failure. Our prevalence of wasting in underfives was similar to that of many other authors (4.4-6.8%) [27, 28, 36]. Comparing the prevalence between the 2 age groups, we noticed that wasting and underweight were more important in children older than 5 years. It is in good similarity to several studies [7, 27, 28, 37]. This can be explained by the frequency and severity of acute complications in our population. Unfortunately, correlations between these complications and the frequency of undernutrition were beyond the scope of the current study. Stunting was the most common form of undernutrition in our population. This is probably related to the chronicity of SCD and the cumulative long-term effect of deficits and complications. It is concordant with several other studies that stunting is the major form of undernutrition observed in children with SCD [38, 39]. In a study carried out in Kinshasa, the number of severe sickle cell crises (more than two per year), the age at the first blood transfusion of less than 12 months, and the number of blood transfusions of more than three per patient was significantly associated with an increased risk of stunting [25].

Apart from the growth deficit, the association between SCD and overweight/obesity has become of interest these last years. We found a prevalence of 3.3%, with children under five being more concerned (5.4%) than children over 5 years (2.2%). Our findings were similar to that of Ukoya and Akodu [27, 40] in Nigeria (5.1 and 2.5% respectively) but much lower than those reported by Chawla [34] in New England (22.4%). This difference can be explained by the better level of management of SCD in developed countries where chronic transfusions, blood exchange, Hydroxyurea, and oral Glutamine are easily used and had been proven to increase BMI in SCD [41]. These therapies are sparsely available in Cameroon and other sub-Saharan African countries.

In our study, the curves of distribution of the indices weight for age, BMI for age, and height for age of less than 5 years of our population did not appear to show any major difference when we compared them to the curve of distribution of a normal population according to WHO. This was already reported by Ngo Um in a previous study in Cameroon [36]. The high prevalence of overweight/obese in high-income countries emphasizes the question of using foreign standards growth curves in normal children to identify impaired growth in children with sickle cell disease from Africa. A study evaluating the growth of pygmies using WHO curves reinforces the fact that ethnic-specific growth curves are better than using a one size fits all approach (WHO curves) that can give us erroneously wrong estimations of growth retardation [42]. However, the distribution curves of the weight-for-age, BMI-for-age, and height-for-age indices of children aged 5-19 clearly showed us a delay in stature and weight. This finding is consistent with that reported by Animasahun et al. and Cox et al., who revealed a pattern of an increasing deficit with increasing age; with boys being more severely affected than girls [7, 9]. In a recent study, age, sex, country, SCD phenotype and parents’ level of education showed that growth failure in patients with SCD was most frequent in adolescents and was associated with parents’ having only primary education and with icterus [43]. The level of education was not evaluated as a possible factor linked to undernutrition in this study.

This study has some limitations. Firstly, although the growth curves used in the current study were based on WHO standards which may not to be appropriate for developing countries like Cameroon [44, 45], this was the only standardised comparable tool in our context. We suggest development of standardised tools to evaluate anthropometric indices in this vulnerable population. Secondly, patients were recruited from the pool of ill sickle cell patients hospitalized or coming for outpatient consultations, making selection bias a plausible methodological flaw. Thirdly, the study was conducted in a single urban center and not a community. This suggests that patients are more learned and wealthier than people living in rural communities and therefore less at risk of malnutrition constituting a selection bias. Fourthly, the study is not a comparative study between the anthropometric parameters of sickle cell children with the normal children in our setting. Also, other limitations include the fact that this study didn’t look at children’s eating practices and behaviors, which are highly dependent on ethnicity. Douala, being a cosmopolitan city, there is quite a large mix of ethnicities in the city. Furthermore, we didn’t look at other aspects of malnutrition (micronutrients, macronutrients). Lastly, conclusive statements can hardly be made on our findings since this study was purely descriptive. The apparent trend differences in the prevalence of major forms of malnutrition based on sociodemographic parameters and others need to be confirmed by robust methodological designs and statistical analyses.

## Conclusion

The present study shows that 9% of children above 5 years with SCD are stunted. The growth deficit increases with age, being more important for boys than girls over 5 years. Anthropometric parameters are stackable with WHO norms for underfives but not for children over five, emphasizing the question of the use that norms for this age group.

## Data Availability

The datasets generated and/or analysed during the current study are not publicly available due to hospital administrative and legal restrictions but are available from the corresponding author on reasonable request.

## References

[1] Steensma DP, Kyle RA, Shampo MA (2010). Walter Clement noel—first patient described with sickle cell disease. Mayo Clin Proc.

[2] Nader E, Romana M, Connes P (2020). The red blood cell—inflammation vicious circle in sickle cell disease. Front Immunol.

[3] Sickle Cell Disease. WHO Reg. Off. Afr. Available from: https://www.afro.who.int/health-topics/sickle-cell-disease [cited 8 May 2022].

[4] Ama Moor VJ, Pieme CA, Chetcha Chemegne B, Manonji H, Njinkio Nono BL, Tchoula Mamiafo C (2016). Oxidative profile of sickle cell patients in a Cameroonian urban hospital. BMC Clin Pathol.

[5] Sedrak A, Kondamudi NP (2022). Sickle Cell Disease. StatPearls.

[6] Al-Saqladi AWM, Bin-Gadeen HA, Brabin BJ (2010). Growth in children and adolescents with sickle cell disease in Yemen. Ann Trop Paediatr.

[7] Cox SE, Makani J, Fulford AJ, Komba AN, Soka D, Williams TN (2011). Nutritional status, hospitalization and mortality among patients with sickle cell anemia in Tanzania. Haematologica.

[8] Shongo MYP, Mukuku O, Mutombo AM, Lubala TK, Ilunga PM, Sombodi WU (2015). Profil hématologique et nutritionnel du drépanocytaire homozygote SS âgé de 6 à 59 mois à Lubumbashi, République Démocratique du Congo. Pan Afr Med J.

[9] Animasahun B, Ogunkunle O, Njokanma O, Temiye E, Izuora A (2011). The influence of socioeconomic status on the hemoglobin level and anthropometry of sickle cell anemia patients in steady state at the Lagos University teaching hospital. Niger J Clin Pract.

[10] Das S, Gulshan J (2017). Different forms of malnutrition among under five children in Bangladesh: a cross sectional study on prevalence and determinants. BMC Nutr.

[11] World Health Organization, United Nations Children’s Fund (UNICEF), World Bank (2021). Levels and trends in child malnutrition: UNICEF / WHO / the World Bank Group joint child malnutrition estimates: key findings of the 2021 edition.

[12] Nartey EB, Spector J, Adu-Afarwuah S, Jones CL, Jackson A, Ohemeng A (2021). Nutritional perspectives on sickle cell disease in Africa: a systematic review. BMC Nutr.

[13] Cochran WG (1977). Sampling techniques.

[14] de Onis M, Onyango AW, Borghi E, Siyam A, Nishida C, Siekmann J (2007). Development of a WHO growth reference for school-aged children and adolescents. Bull World Health Organ.

[15] World Health Organization (2009). WHO child growth standards: growth velocity based on weight, length and head circumference: methods and development.

[16] World Health Organization (2009). WHO AnthroPlus for personal computers Manual.

[17] de Onis M, Onyango AW, Van den Broeck J, Chumlea WC, Martorell R (2004). Measurement and standardization protocols for anthropometry used in the construction of a new international growth reference. Food Nutr Bull.

[18] Lukusa Kazadi A, Ngiyulu RM, Gini-Ehungu JL, Mbuyi-Muamba JM, Aloni MN (2017). Factors associated with growth retardation in children suffering from sickle cell Anemia: first report from Central Africa. Anemia.

[19] Growth reference 5-19 years - Application tools;Available from: https://www.who.int/tools/growth-reference-data-for-5to19-years/application-tools [cited 30 Jul 2022].

[20] Metcalfe C (2001). Biostatistics: A Foundation for Analysis in the Health Sciences. 7th ed. Wayne W. Daniel, Wiley, 1999. No. of. pages: xiv+755+appendices. Price: £28.95. ISBN 0-471-16386-4. Stat Med.

[21] Dean AG, Sullivan KM, Soe MM (2013). OpenEpi: Open Source Epidemiologic Statistics for Public Health, Version 3.01.

[22] Musa TH, Musa HH, Ali EA, Musa NE (2020). Prevalence of malnutrition among children under five years old in Khartoum state, Sudan. Pol Ann Med.

[23] Akenji TN, Sumbele I, Mankah EN, Njunda AL, Samje M, Kamga L (2008). The burden of malaria and malnutrition among children less than 14 years of age in a rural village of Cameroon. Afr J Food Agric Nutr Dev.

[24] Penda CI, Moukoko ECE, Nolla NP, Evindi NOA, Ndombo PK (2018). Malnutrition among HIV infected children under 5 years of age at the Laquintinie hospital Douala, Cameroon. Pan Afr Med J.

[25] Lukusa Kazadi A, Ngiyulu RM, Gini-Ehungu JL, Mbuyi-Muamba JM, Aloni MN (2017). Factors associated with growth retardation in children suffering from sickle cell Anemia: first report from Central Africa. Anemia.

[26] Esezobor CI, Akintan P, Akinsulie A, Temiye E, Adeyemo T (2016). Wasting and stunting are still prevalent in children with sickle cell anaemia in Lagos, Nigeria. Ital J Pediatr.

[27] Ukoha O, Emodi I, Ikefuna A, Obidike E, Izuka M, Eke C (2020). Comparative study of nutritional status of children and adolescents with sickle cell anemia in Enugu, Nigeria. Niger J Clin Pract.

[28] Boadu I, Ohemeng A, Renner LA (2018). Dietary intakes and nutritional status of children with sickle cell disease at the Princess Marie Louise Hospital, Accra – a survey. BMC Nutr.

[29] Zemel BS, Kawchak DA, Ohene-Frempong K, Schall JI, Stallings VA (2007). Effects of delayed pubertal development, nutritional status, and disease severity on longitudinal patterns of growth failure in children with sickle cell disease. Pediatr Res.

[30] GHO | Global Health Observatory Data Repository (African Region) | Prevalence of thinness among children and adolescents, BMI < -2 standard deviation below the median, crude - Estimates by country, among children 5-19 years. WHO. Available from: https://apps.who.int/gho/data/node.main-afro.NCDBMIMINUS2C? [cited 9 May 2022].

[31] Barden EM, Zemel BS, Kawchak DA, Goran MI, Ohene-Frempong K, Stallings VA (2000). Total and resting energy expenditure in children with sickle cell disease. J Pediatr.

[32] Enyuma CO, Anah MU, Pousson A, Olorunfemi G, Ibisomi L, Abang BE (2019). Patterns of paediatric emergency admissions and predictors of prolonged hospital stay at the children emergency room, University of Calabar Teaching Hospital, Calabar, Nigeria. Afr Health Sci.

[33] Nunlee-Bland G, Rana SR, Houston-Yu PE, Odonkor W (2004). Growth hormone deficiency in patients with sickle cell disease and growth failure. J Pediatr Endocrinol Metab.

[34] Chawla A, Sprinz PG, Welch J, Heeney M, Usmani N, Pashankar F (2013). Weight status of children with sickle cell disease. Pediatrics.

[35] Zemel BS, Kawchak DA, Fung EB, Ohene-Frempong K, Stallings VA (2002). Effect of zinc supplementation on growth and body composition in children with sickle cell disease. Am J Clin Nutr.

[36] Ngo Um SS, Seungue J, Alima AY, Mbono R, Mbassi H, Chelo D (2019). A cross sectional study of growth of children with sickle cell disease, aged 2 to 5 years in Yaoundé, Cameroon. Pan Afr Med J.

[37] Islam MR, Moinuddin M, Ahmed A, Rahman SM (2021). Association of sickle cell disease with anthropometric indices among under-five children: evidence from 2018 Nigeria demographic and health survey. BMC Med.

[38] Mitchell MJ, Carpenter GJO, Crosby LE, Bishop CT, Hines J, Noll J (2009). Growth status in children and adolescents with sickle cell disease. Pediatr Hematol Oncol.

[39] Mabiala-Babela JR, Massamba A, Tsiba JB, Moulongo JG, Nzingoula S, Senga P (1990). Body composition in negro African children suffering from sickle cell disease. A mixed cross-sectional longitudinal study in Brazzaville, Congo. Bull Soc Pathol Exot.

[40] Akodu SO, Diaku-Akinwumi IN, Njokanma OF (2012). Obesity—does it occur in Nigerian children with sickle cell Anemia. Pediatr Hematol Oncol.

[41] Galadanci NA, Sohail M, Akinyelure OP, Kanter J, Ojesina AI (2022). Treatment-related correlates of growth in children with sickle cell disease in the DISPLACE cohort. J Pediatr Hematol Oncol.

[42] Funk SM, Guerra BP, Ickowitz A, Poni NA, Abdou MA, Sibama YH (2019). WHO child growth standards for pygmies: one size fits all? Scientific Communication and Education.

[43] Alexandre-Heymann L, Dubert M, Diallo DA, Diop S, Tolo A, Belinga S (2019). Prevalence and correlates of growth failure in young African patients with sickle cell disease. Br J Haematol.

[44] Wang Y, Moreno LA, Caballero B, Cole TJ (2006). Limitations of the current World Health Organization growth references for children and adolescents. Food Nutr Bull.

[45] Ghafuri DL, Abdullahi SU, Jibir BW, Gambo S, Bello-Manga H, Haliru L (2020). World Health Organization’s growth reference overestimates the prevalence of severe malnutrition in children with sickle cell Anemia in Africa. J Clin Med.

